# Effects of GLP-1 Agonists on Patients with Hidradenitis Suppurativa: A Systematic Review

**DOI:** 10.3390/jcm15082909

**Published:** 2026-04-11

**Authors:** Annik Caliezi, Aref Hosseini, Ronald Wolf, Seyed Morteza Seyed Jafari

**Affiliations:** 1 Department of Dermatology, Inselspital, Anna-Seiler-Allee 33, 3010 Bern, Switzerland; 2 Institute of Pharmacology, University of Bern, Rosenbühlgasse 27, 3010 Bern, Switzerland

**Keywords:** effects, glucagon like peptide 1 receptor agonists, hidradenitis suppurativa, quality of life, cardiovascular risk

## Abstract

**Background:** Hidradenitis suppurativa (HS) is a chronic inflammatory skin disease, which presents with painful nodules, abscesses and sinus tracts. Patients suffer from pain, drainage and worsening of mental health and quality of life. Treatment is often difficult. HS is typically associated with obesity and metabolic syndrome; thus, antidiabetics, especially GLP-1 agonists, present a potential therapy option. The aim of this review was to analyze the effects of GLP-1 agonists on patients with HS, including on their cardiovascular risk and quality of life. **Methods:** A literature search was conducted on Embase and PubMed, yielding 300 papers, of which 10 were used for this review. **Results:** HS patients using GLP-1 agonists showed improved clinical course with less pain and suppuration. Further, patients’ quality of life and mental health improved and their cardiovascular risk was reduced. Inflammatory parameters showed no significant changes. Patients receiving a higher drug dose of GLP-1 agonists were more likely to show clinical improvement. A reduction in weight or BMI did not correlate with improvements in Hurley stage, pain or depression. Hence, HS patients could be treated with GLP-1 agonists. **Conclusions:** Therefore, whether patients’ improvement is due to weight loss, or other mechanisms, i.e., GLP-1 agonists’ anti-inflammatory properties, remains to be determined in further studies.

## 1. Introduction

Hidradenitis suppurativa (HS) is a chronic inflammatory skin disease where patients suffer from painful nodules, abscesses and sinus tracts typically located in intertriginous areas [1]. Patients experience pain, malodorous drainage and a great negative impact on their mental health [2,3,4]. All these factors together cause HS patients to have one of the worst quality of life impairments among dermatologic patients [5].

The etiology of HS is not fully understood, but three key events are known in its pathogenesis: follicular occlusion and dilatation, follicular rupture with subsequent acute and severe immune response, and chronic inflammation with sinus tract formation [2,6]. Possible causes or contributing factors include genetic mutations, upregulation of certain cytokines, alteration of local skin microbiome, and physical, psychological and environmental factors such as smoking and obesity [2,6]. Obesity may contribute to the development of HS through the pro-inflammatory state, promoted by the production of pro-inflammatory adipokines and suppression of anti-inflammatory ones by adipose tissue [7]. In addition, the accumulation of fat and insulin resistance are associated with a change in immune cell composition and function within adipose tissue [8]. Apart from obesity, HS could be strongly correlated with metabolic syndrome and aspects thereof [1,9]. Up to 50% of HS patients suffer from metabolic syndrome, with dyslipidemia, obesity and hyperglycemia being most associated with HS [1,9,10]. Consequently, HS patients have an elevated risk for complications of metabolic syndrome, like atherosclerosis, cardiovascular events, type 2 diabetes (T2DM), and non-alcoholic fatty liver disease (NAFLD) [1,10,11].

Until now, HS has still been difficult to manage and treatment often remained unsatisfying [1]. Depending on the stage of HS and the extent of disease, different therapeutic approaches and combinations thereof are utilized [1,12]. In addition to clinical assessment, radiological modalities such as sonography, color Doppler, or MRI could be helpful [13,14]. Besides pharmaceutical therapies, surgical interventions may be helpful for individuals. Acute abscesses are treated with incision and drainage, whereas sinus tracts and tunnels require complete deroofing or complete excision [1]. Though medication and surgery are the primary treatments for HS, adjuvant therapies like pain management and mental health support, as well as lifestyle adjustments like weight loss and cessation of smoking, must be considered [1]. The problem with these current therapy options is their response rate and long-term success. Topical and systemic antibiotics are first-line treatments, with the first having little data about their use and a proneness to resistance, while the second show a response rate of about 48% [1,15]. Biologics such as adalimumab show a response in half of patients with the risk of loss of efficacy [1,16]. Surgical procedures have high relapse rates and are linked with worse anxiety and depression [1].

Given that obesity and metabolic syndrome are closely associated with HS, weight loss is recommended, which may now be supported with GLP-1 agonists. GLP-1 agonists are pharmaceuticals primarily utilized in patients with T2DM or obesity, given that they stimulate insulin secretion and cause delayed gastric emptying and increased satiety, which promotes weight loss and lowering of serum glucose levels and HbA1c [17]. Commonly utilized drugs are Semaglutide, Liraglutide and Dulaglutide. Many new drugs are currently being researched, i.e., *Maridebart cafraglutide*, which is injected once monthly, as shown in a Phase 2 study [18]. In previous case reports like Jennings et al., GLP-1 agonists have been observed to improve pain, QoL and Hurley stage; therefore, this review seeks to summarize the effects of GLP-1 agonists on HS patients [19].

## 2. Materials and Methods

**Figure 1:**
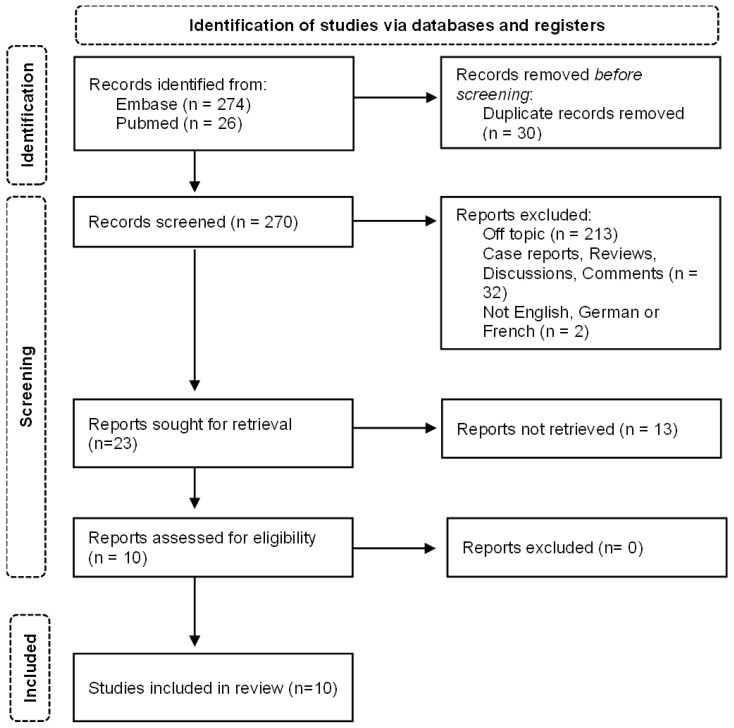
Study design.

We conducted a literature search on Embase (23 October 2025) and PubMed (26 October 2025) using the following key words and variations or abbreviations of them: “Hidradenitis suppurativa”, “acne inversa”, “Verneuil’s disease”, “pyoderma fistulans significa”, “apocrine acne”, “Incretin mimetic”, “glucagon like peptide 1 agonist”, “glucagon like peptide 1 analogue”. In addition, the drug names of 15 GLP-1 agonists that are currently approved or being evaluated in clinical trials, such as “semaglutide” or “efpeglenatide”, and brand names of 18 medications currently on the market, e.g., “Mounjaro” or “Xultophy”, were used to extend the search. “Hidradenitis suppurativa”, “glucagon like peptide”, “glucagon like peptide 1”, “glucagon like peptide receptor”, “glucagon like peptide 1 receptor”, “glucagon like peptide receptor agonist”, “glucagon like peptide 1 receptor agonist”, “glucagon like peptide derivative” were used as Emtree and MeSH terms, together with those beforementioned drug names, which are listed as Emtree/MeSH terms (complete search strings in Appendix A). No published search filters were applied, and no previously existing search strategies were used. Searches were rerun and improved multiple times, and updates via email were utilized. This search yielded 274 publications on Embase and 26 on PubMed. Papers that were off-topic, not available in English, German or French, or not accessible to the authors were excluded. Reviews, comments, discussions and case reports were excluded. The latter was due to the limited comparability and scientific evidence gained. Given the large number of papers that were off-topic or only mentioned HS or GLP-1 agonists, we were left with 10 papers, all of which were analyzed (Figure 1, Table 1). Data extraction and synthesis of results were conducted systematically by the first author and supervised by the last author. Quantitative synthesis was not feasible due to the heterogeneity of endpoints and designs of the original studies. This review adheres to the PRISMA guidelines for systematic reviews, and was not registered (PRISMA 2020 Checklist; PRISMA-S Checklist).

## 3. Results

### 3.1. Skin and HS

In multiple studies, clinical evaluations of HS were performed under treatment with GLP-1 agonists [22,23,26,27,29]. The utilized objective measures, HS-PGA and Hurley score, improved significantly (*p* < 0.001 [22]; mean Hurley 2.6 ± 0.5 to 1.1 ± 0.3, *p* = 0.002 [27]). A case series with seven patients observed Hurley scores to improve in three cases, though one of them had bariatric surgery done and another started secukinumab [29]. Pain and suppuration were noted to have significantly improved in the majority of patients taking GLP-1 agonists (Pain: 52.4% of patients [23], *p* < 0.001 [22], *p* = 0.003 [27]; Suppuration: 61.9% of patients [23], *p* = 0.001 [22]). The majority of patients reported fewer flares and less new lesions (61.9%, resp. 66.7%) [23]. One study found the decreasing frequency of flares to be significant (*p* < 0.001 [22]) in contrast to another one (*p* = 0.38 [26]). Some patients experienced less itching and less odor (47.6%, resp. 42.9%) [23]. Hill and Bordeaux [24] investigated how Semaglutide influenced overweight HS patients’ utilized HS resources. They found that the use of antibiotics and steroids, as well as ER visits, decreased after beginning the treatment with Semaglutide, while the use of biologics did not significantly change (risk ratio: 0.758, CI: 0.732–0.785/RR: 0.839, CI: 0.811–0.868/RR: 0.715, CI: 0.681–0.751/RR: 0.983, CI: 0.862–1.122) [24].

One study looked back to before the diagnosis of HS was made, and found that diabetic patients treated with a GLP-1 agonist were less likely to ever develop HS than those without GLP-1 agonists (OR = 0.61, 95% CI: 0.48–0.76) [20].

### 3.2. Metabolic Syndrome and HS

It is widely accepted that obesity is an important risk factor for HS [30]. The four studies that looked at patients’ weight observed that the majority of patients lost weight while being on GLP-1 agonists [23,26,27,28]. BMI decreased significantly in two studies, but insignificantly in a third (*p* < 0.001 [22]; *p* = 0.001 [27]; *p* = 0.48 [26]). Besides obesity, other aspects of the metabolic syndrome improved: systolic and diastolic blood pressure decreased (both *p* = 0.001), LDL-cholesterol rose insignificantly and HDL-cholesterol declined insignificantly (*p* = 0.9, *p* = 0.06) [27]. Two studies compared blood sugar in HS patients without diabetes before and during treatment with Liraglutide and Semaglutide. The HbA1c levels lowered significantly when taking Semaglutide but not Liraglutide, and the fasting glucose did not change significantly with either therapy (*p* = 0.03 [26], *p* = 0.07 [27]; *p* > 0.99 [26], *p* = 0.3 [27]). Notably, insulin decreased significantly in the study testing Liraglutide (*p* = 0.005) [27].

### 3.3. Cardiovascular Risk and HS

Two articles studied cardiovascular outcomes in HS patients while they were medicated with GLP-1 agonists. Significantly fewer acute myocardial infarctions, strokes, and cardiovascular and cerebrovascular composite events were observed in patients taking Semaglutide (*p* = 0.009, RR 0.76 [CI 0.62–0.94]; *p* = 0.003, RR 0.69 [CI 0.54–0.88], *p* < 0.001; RR 0.73 [CI 0.61–0.87]) [25]. The survival without any of these events was also significantly better in Semaglutide users (AMI: *p* < 0.001, stroke: *p* < 0.001, composite: *p* < 0.001) [25]. Another study found the odds for myocardial infarction and cerebrovascular events to be significantly diminished after 5, 10 and 20 years ([20 y for MI: aOR = 0.69 (95% CI= 0.63–0.77)], [20 y for CV events: aOR = 0.73 (95% CI= 0.66–0.80)]) [21]. This reduction in odds was also described regarding ischemic heart disease, heart failure, atherosclerosis and percutaneous coronary intervention [21].

### 3.4. Quality of Life and Mental Health in Patients with HS

All three studies that looked at quality of life (QoL), measured by the Dermatologic Life Quality Index (DLQI), recognized it to improve significantly (*p* < 0.001 [22], *p* = 0.04 [27], *p* = 0.001 [26]). Depressive symptoms, measured with Beck’s Depression Inventory (BDI), improved as well, as did binge eating and emotional eating (*p* = 0.007, *p* = 0.008, *p* = 0.005) [27]. A majority of patients also noted less impact of HS on their daily life while taking GLP-1 agonists (59.1%) [23].

### 3.5. Laboratory Analyses

One study found no correlation between the observed reduction in BMI and the improvement in Hurley stage, VAS for pain, BDI, or any change in inflammatory parameters [27]. Another proposed mechanism of GLP-1 agonists is that they reduce inflammation and, thus, improve HS [20,31]. Lyons et al. measured systemic inflammatory parameters and observed CRP, white cell count, neutrophils, lymphocytes and neutrophil-to-lymphocyte ratio to decrease insignificantly (*p* = 0.95, *p* > 0.99, *p* > 0.99, *p* > 0.99, *p* > 0.99) [26]. Another study noticed a significant reduction of ultrasensitive CRP levels (*p* = 0.04) but not of ferritin (*p* = 0.2) [27].

On average, patients showed mild hyperhomocysteinaemia before treatment with Liraglutide, which diminished significantly (cut-off 15 µmol/L; before 16.2 µmol/L (±2.9), after 13.3 µmol/L (±3.0) *p* = 0.005) [27,32]. Laboratory analyses were performed in only 44 patients and no standardized markers were tested; thus, no definite conclusions may be drawn.

### 3.6. Medication, Dosage, Responders, Time Frame

The most commonly used medication was Semaglutide (mentioned in eight studies), then Liraglutide (five), Dulaglutide (four) and Tirzepatide (two), while Albiglutide, Lixisenatide and Exenatide were mentioned only once. No comparisons were made among these substances.

Dosages were declared in three of ten articles: Liraglutide was used at 3 mg weekly [27] and Semaglutide once at an average dose of 0.8 mg (SD 0.4) weekly [26] and once at an average of 1.36 mg (SD 0.86) weekly [28]. In the latter study, responders to Semaglutide were retrospectively compared to non-responders. Response was defined through need for rescue therapies, change in lesion counts, and clinical reevaluation by the same dermatologist. It was found that responders received a significantly higher mean dose of Semaglutide and this dose-dependent effect was confirmed by their logistic regression model (6 month: responders: 1.3 mg ± 0.9, non-responders: 0.8 mg ± 0.6; *p* = 0.02; logistic regression of 6 month dose *p* = 0.045) [28]. Notably, there were trends towards men and smokers showing higher response rates, but a higher dosage was the only significant difference between the two groups [28]. Disease severity (*p* = 0.55), prior disease course (*p* = 0.9) and weight loss were all insignificantly different between responders and non-responders [28].

Duration of treatment was analyzed scarcely, ranging from three to 17 months [23,26,27,28,29]. Gupta et al. detected no association between the duration of treatment and HS-specific health [23].

## 4. Discussion

**Figure 2:**
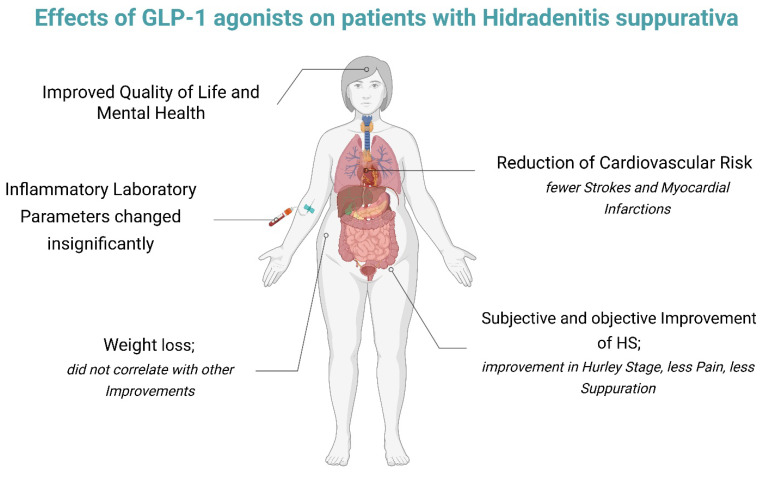
Possible effects of GLP-1 agonists on patients with hidradenitits suppurativa. Created in BioRender. Caliezi, A. (2026) https://BioRender.com/qqesyea (accessed on 20 March 2026).

GLP-1 agonists have received a great amount of attention in medicine in the last few years. In dermatology, they have been reported to improve psoriasis and other inflammatory cutaneous diseases [33,34]. Due to the association between HS and metabolic syndrome, and because of observations of clinical improvement of HS in case reports, GLP-1 agonists are an intriguing new therapeutic option. This review has found that GLP-1 agonists can help alleviate HS disease burden, including reductions in pain, suppuration, flares, quality of life impairment, and amelioration of mental health (Figure 2). Given that obesity is a risk factor for HS, it is plausible that GLP-1 agonists driven weight loss could be a reason for improvement [35]. This improvement may be mediated by reduced friction and humidity in intertriginous areas, which promote follicular damage and local inflammation, as well as by reduced adipose tissue mass with consequent decrease in adipose-tissue-derived pro-inflammatory adipokines and cytokines [7,27]. Surprisingly though, one study found no correlation between the reduction in BMI and the improvement in clinical HS stage, pain, depression score or the evolution of inflammatory parameters. Given that this finding is supported by only one study, the extent to which the improvement is attributable to weight loss remains unclear. Another explanation for the improvement could be GLP-1 agonists’ anti-inflammatory qualities [36,37]. This is supported by research observing cytokines important in HS, like TNF-α and IL-6, to diminish when treated with GLP-1 agonists [31,38,39]. However, improvements extend beyond physical symptoms, as reductions in disease burden may directly translate into better mental health and quality of life. Alternatively, improvements in these domains may also be driven by reduced systemic inflammation, as growing evidence links depression and anxiety to chronic inflammatory states [40,41]. Whether decreased inflammation is the reason for improvement remains speculative, given that only ultrasensitive CRP was observed to decrease significantly, while all other inflammatory parameters were insignificantly changed. But this insignificance might be of limited statistical power, given that inflammatory parameters were only measured in 44 patients altogether.

HS patients using GLP-1 agonists experienced a significant reduction in the risk of cardiovascular disease, cardiovascular events, and mortality. This benefit was also observed when patients with psoriasis were treated with GLP-1 agonists [33]. This effect may be explained by weight loss and decreased blood pressure observed in prior studies, or improved glycemic control, given that most patients included in the cardiovascular analyses received GLP-1 agonists for diabetes. Another theory, supported by the preexisting literature states the persistent systemic inflammation in HS to be the reason for higher cardiovascular risk as pro-inflammatory cytokines found in HS contribute to atherosclerosis and thrombosis [42,43]. A third possibility is that a reduced cardiovascular risk may be associated with decreased homocysteine levels observed during GLP-1 receptor agonist therapy given that hyperhomocysteinaemia is an established risk factor for cardiovascular disease [44]. Whether cardiovascular risk improved due to the amelioration of HS or solely as a consequence of the effects of GLP-1 agonists on metabolic syndrome remains inconclusive. As of now, recommendations for cardiovascular risk minimization in HS patients include screening for comorbidities, like obesity, dyslipedaemia, diabetes and nicotine consumption, and consequent prevention, respectively, for treatment of these comorbidities [42]. In addition to these common preventative measures, systemic anti-inflammatory treatment is currently being discussed and researched, but is not yet understood well enough [42,45,46].

Altogether, GLP-1 agonists might be a promising adjunctive therapy option for HS patients. In comparison with current long-term treatment options like biologics, they seem to have a similar response rate, ranging between 42% and 68%, with biologics at about 50% [1,16,22,23,28,29]. The only significant difference between responding patients and non-responders was the significantly higher dose of Semaglutide administered to responders. Based on the hypothesis that GLP-1 agonists have a positive effect on HS, we suggest the use of medication that has shown its benefits in some of the studies, namely Semaglutide and Liraglutide. We suggest Semaglutide to be used at an end dose of 2.4 mg weekly, in accordance with general recommendations for weight loss [47]. For Liraglutide, we recommend an end dose of 3 mg weekly, as this worked well in Nicolau et al.’s work and is again in accordance with guidelines for weight loss [48]. Duration of treatment was observed not to be associated with HS-specific health by Gupta et al.; therefore, we orientate ourselves to the WHO guidelines for obesity, which state to use GLP-1 agonists long-term [49]. We use guidelines for obesity, given that we cannot yet rule out weight loss to be the reason for improvement. Currently, we recommend using GLP-1 agonists primarily in obese HS patients, given the small amount of clinical experience and research, and even less so about non-obese patients.

Furthermore, artificial intelligence (AI) is showing a steady improvement in diagnostic and therapeutic capabilities and, therefore, presents a potential future tool for screening and education of HS and other skin diseases [50,51]. Thus, it may aid doctors to evaluate GLP-1 agonist treatment in HS in the future, but much more research is needed. Limitations of this review include the small number of studies. Furthermore, clinical trials had few participants, often there were no controls, and other treatments were not declared in detail and changed during the observation periods; thus, the causality of improvements is difficult to establish. Most studies were retrospective cohorts; thus, patients most likely received GLP-1 agonists because of obesity or diabetes, not HS. Another issue is the lack of standardized questionnaires, measures, and outcomes, which makes the extraction of general statements difficult. Given this substantial risk of bias in all analyzed studies, our results must be interpreted with caution (Table S1). Future research should use standardized measures, attempt large-scale randomized clinical trials with placebo control groups, and look into the effects of GLP-1 agonists in non-obese patients too. Furthermore, additional studies are warranted to elucidate the mechanisms underlying the observed improvements, as current explanations, like weight loss and anti-inflammatory properties, remain largely speculative.

## 5. Conclusions

HS patients treated with GLP-1 agonists have improved objective disease, less pain, suppuration and flares, as well as better mental health and quality of life. Additionally, they have a lower risk of cardiovascular diseases or events and a lower cardiovascular mortality rate. Proposed mechanisms for this benefit are weight-loss-related mechanical and metabolic effects, as well as the anti-inflammatory properties of GLP-1 agonists. This might suggest that obese HS patients could be treated more generously with GLP-1 agonists. The next step is to perform randomized clinical trials and continue research with prospective studies in obese and non-obese HS patients.

## Figures and Tables

**Table 1:** Analyzed articles.

Author	Design	HS+ GLP-1 RA (* Controls)	Outcomes	Medication	Results
Ching et al. 2025 [20]	Retrospective cohort study	601 HS, + 1601 Psoriasis (*0)	Risk of HS or Psoriasis	Albiglutide, Dulaglutide, Exenatide, Liraglutide, Lixisenatide, Semaglutide	- T2DM patients taking GLP-1 agonists have lower risk for diagnosis of HS (OR = 0.61, 95% CI, 0.48–0.76, *p* < 0.001) - T2DM patients taking GLP-1 agonists have lower risk for diagnosis of Psoriasis (OR = 0.41, 95% CI, 0.35–0.48, *p* < 0.001)
Chou et al. 2026 [21]	Retrospective cohort study	19,920 (*19,920)	Odds for cardiovascular events in HS patients taking GLP-1 agonists	Any GLP-1 RA	- After 5/10/20 years, GLP-1RA users with HS have significantly lower odds of cerebrovascular disease (20 y: aOR 0.73 [0.66–0.80]), AMI (20 y: aOR 0.69 [0.63–0.77]), ischemic heart disease (20 y: aOR 0.77 [0.72–0.81]), heart failure (20 y: aOR 0.71 [0.66–0.75]), atherosclerosis (20 y: aOR 0.75 [0.68–0.81]), PCI (20 y: aOR 0.35 [0.20–0.59])
Gouvrion et al. 2025 [22]	Retrospective cohort study	66 (*0)	HS-PGA, Flare frequency, NRS pain, NRS Suppuration, DLQI	Semaglutide, Dulaglutide, Liraglutide	- Reduction in HS-PGA in majority of patients (6 months 54%: *p* < 0.001) - Flares, NRS-Pain, NRS-Suppuration reduced in majority of patients (6 mo: 60%: *p* < 0.001; 52%: *p* > 0.001; 53%: *p* = 0.001) - DLQI improved (6 mo: 50%: *p* < 0.001) - Reduction in BMI (6 mo: *p* > 0.001)
Gupta et al. 2025 [23]	Cross-sectional survey study	22 (*0)	Flares, Pain, Suppuration, drainage, lesions, Impact on daily activities	Semaglutide, Tirzepatide, Dulaglutide, Liraglutide	- No association between duration of treatment and change in HS-specific health - 77.3% of patients reported weight loss - 68.2% reported improvement in HS-specific health: Reduction in flares (61.9%), new lesions (66.7%), pain (52.4%), drainage (61.9%), itch (47.6%), and odor (42.9%). - Less impact of HS on daily activities (59.1%)
Hill and Bordeaux 2025 [24]	Retrospective cohort study	6639 (*6639)	Impact of therapy on HS resource utilization	Semaglutide	- Patients on Semaglutide showed less use of antibiotics (RR: 0.758, CI: 0.732–0.785), fewer visits to the ED (RR: 0.715, CI: 0.681–0.751), and less use ofsteroids (RR: 0.839, CI: 0.811–0.868) - No change in use of biologics (RR: 0.983, CI: 0.862–1.122)
Islam et al. 2026 [25]	Retrospective cohort study	14,850 (*193,575)	MACE: acute myocardial infarction, stroke, composite of AMI and stroke	Semaglutide	- Risk in GLP-1 agonist group lower for AMI (*p* = 0.009; RR 0.76 [0.62–0.94]), stroke (*p* = 0.003; risk ratio 0.69 [0.54–0.88]) and composite MACE (*p* < 0.001; risk ratio 0.73 [0.61–0.87]) - Event-free survival higher in GLP-1 group (AMI: *p* < 0.001; hazard ratio [HR: 0.68] [95% CI: 0.55–0.84]; stroke: *p* < 0.001; HR: 0.63 [95% CI: 0.49–0.80]; composite: *p* < 0.001; HR: 0.66 [95% CI: 0.55–0.78])
Lyons et al. 2024 [26]	Retrospective cohort study	30 (*0)	Flares, QoL (DLQI), weight, inflammatory markers, blood sugar	Semaglutide	- Mean frequency of flares reduced insignificantly (*p* = 0.38) - Improvement in mean QoL (*p* = 0.001) - CRP, WCC, Lymphocytes, Neutrophils, neutrophil: lymphocyte ratio decreased (*p* = 0.95, *p* > 0.99, *p* > 0.99, *p* > 0.99, *p* > 0.99) - HbA1c decreased significantly (*p* = 0.03, fasting glucose insignificantly (*p* > 0.99) - Mean weight decreased (*p* < 0.001), BMI sank insignificantly (*p* = 0.48)
Nicolau et al. 2024 [27]	Retrospective proof-of-concept study	14 (*0)	Impact on HS, QoL (DLQI), mental health (BDI), inflammatory markers, cardiovascular risk factors	Liraglutide	- Hurley stage and pain (VAS) decreased (*p* = 0.002, *p* = 0.003) - DLQI and BDI improved (*p* = 0.04, *p* = 0.007) - Binge eating disorder and Emotional eating improved (*p* = 0.008, *p* = 0.008) - Ultrasensitive CRP reduced significantly (*p* = 0.04), Ferritin insignificantly (*p* = 0.2) - Homocysteine decreased (*p* = 0.005) - Weight, BMI, and waist circumference sank (*p* = 0.001, *p* = 0.002, *p* = 0.01). BMI reduction did not correlate with change in Hurley stage, pain, changes in BDI or inflammatory parameters in logistic regression - HbA1c and fasting glucose decreased insignificantly (*p* = 0.07, 0.3) - Insulin decreased (*p* = 0.005) - Decrease in systolic and diastolic BP (*p* = 0.001, *p* = 0.001). Total cholesterol, triglyceride, LDL and HDL decreased insignificantly (*p* = 0.8, *p* = 0.5, *p* = 0.9, *p* = 0.06)
Posada Posada et al. 2025 [28]	Retrospective cohort study	45 total: 27 responders, 18-non responders (*0)	Differences between responders and non-responders	Semaglutide	- 27 patients improved on Semaglutide (measure: change in lesion count, need for rescue therapy, evaluation by dermatologist) - Only significant difference was higher mean dose given to responders (6 months: *p* = 0.02, logistic regression: OR 2.76, 95% CI 1.02–7.48, *p* = 0.045) - Trend towards men and smokers being more respondent (*p* = 0.06; *p* = 0.09). Logistic regression: *p* = 0.1, *p* = 0.08 - HS stage and prior disease course not significant (*p* = 0.55, *p* = 0.9) - Weight loss not significantly different between groups (6 months: *p* = 0.73)
Rames et al. 2025 [29]	Case series	7 (*0)		Dulaglutide (4), Liraglutide (1), Semaglutide (1), Tirzepatide (1)	- Objective improvement in 3 patients: - 1 (Dulaglutide): Improvement in Hurley stage, lesions and inflammatory parameters. Patient got bariatric surgery during interval - 2 (Dulaglutide, Tirzepatide): improvement in Hurley stage and subjective improvement. One patient started Secukinumab during interval

Abbreviations: aOR: adjusted odds ratio, AMI: acute myocardial infarction, BDI: Beck’s Depression Index, BMI: Body Mass Index, BP: blood pressure, CI: 95% confidence interval, CRP: C-reactive Protein, DLQI: Dermatology Life Quality Index, ED: Emergency Department, GLP-1 RA: GLP-1 receptor agonist, HDL: high-density lipoprotein, HS-PGA: HS Physician Global Assessment, LDL: low-density lipoprotein, MACE: Major Adverse Cardiovascular Event, NRS: numeric rating scale, OR: odds ratio, PCI: Percutaneous Coronary Intervention, QoL: quality of life, RR: risk ratio, T2DM: type 2 diabetes, VAS: Visual Analogue Scale, WCC: white cell count, y: years.

## Data Availability

No new data were created or analyzed in this study. Data sharing is not applicable to this article.

## Supplementary Materials

The following supporting information can be downloaded at: https://www.mdpi.com/article/10.3390/jcm15082909/s1, Table S1: Risk of Bias assessment.; PRISMA 2020 Checklist; PRISMA-S Checklist.

## Abbreviations

The following abbreviations are used in this manuscript (in order of appearance):

HS	Hidradenitis suppurativa
T2DM	Type 2 diabetes mellitus
NAFLD	Non-alcoholic fatty liver disease
GLP-1 agonists	Glucose-like-peptide-1 agonists
HS-PGA	HS-Physician Global Assessment
RR	Risk ratio
CI	Confidence interval
ER	Emergency Room
BMI	Body Mass Index
(A)MI	(Acute) Myocardial Infarction
aOR	Adjusted odds ratio
QoL	Quality of Life
DLQI	Dermatology Life Quality Index
BDI	Beck’s Depression Index
VAS	Visual Analogue Scale
SD	Standard deviation
AI	Artificial intelligence

## Appendix A

### Appendix A.1. Complete Search String Embase

(exp Hidradenitis Suppurativa/ OR (hidradenitis suppurativ* OR suppurative hidradenitis OR HS OR acne inversa OR verneuil* disease OR inverse acne OR pyodermia fistulans significa OR apocrine acne).ti,ab,kw.) AND ((Incretin mimetic* OR glucagon like peptide receptor agonist OR glucagon like peptide 1 receptor agonist OR GLP receptor agonist OR GLP1 receptor agonist OR GLP 1 receptor agonist OR Glucagon like peptide RA OR Glucagon like peptide 1 RA OR GLP RA OR GLP1 RA OR GLP 1 RA OR GLP1R agonist OR glucagonlike peptide OR glucagonlike peptide 1 OR glucagonlike peptide receptor OR glucagonlike peptide receptor agonist OR glucagonlike peptide RA OR glucagonlike peptide 1 receptor OR glucagonlike peptide 1 receptor agonist OR glucagonlike peptide 1 RA OR glucagonlike peptide analog* OR glucagonlike peptide 1 analog* OR glucagon like peptide analog* OR glucagon like peptide 1 analog* OR GLP1 analog* OR GLP 1 analog* OR Semaglutid* OR exenatid* OR liraglutid* OR albiglutid* OR tirzepatid* OR lixisenatid* OR dulaglutide* OR Ozempic OR Rybelsus OR wegovy OR Exendin?4 OR Byetta OR Bydureon OR Saxenda OR Victoza OR exenatide* extended release OR Xultophy OR Eperzan OR Mounjaro OR Zepbound OR Lyxumia OR Adlyxin OR LixiLan OR suliqua OR Trulicity OR efpeglenatid* OR pegapamodutid* OR cotadutid* OR danuglipron OR retatrutid* OR petrelintid* OR mazdutid* OR orforglipron).ti,ab,kw. OR (exp glucagon like peptide 1/ OR exp glucagon like peptide 1 receptor agonist/ OR exp glucagon like peptide/ OR exp glucagon like peptide receptor agonist/ OR exp exendin 4/ OR exp glucagon like peptide 1 receptor/ OR exp glucagon like peptide receptor/ OR exp dulaglutide/ OR exp semaglutide/ OR exp liraglutide/ OR exp albiglutide/ OR exp tirzepatide/ OR exp danuglipron/ OR exp mazdutide/ OR exp insulin glargine plus lixisenatide/ OR exp lixisenatide/ OR exp pegapamodutide/ OR exp cotadutide/ OR exp retatrutide/ OR exp petrelintide/ OR exp orforglipron/ OR exp glucagon like peptide 1 derivative/))

### Appendix A.2. Complete Search String PubMed

((((((((((((((((((glucagon like peptide 1[MeSH Terms]) OR (glucagon like peptide 1 receptor[MeSH Terms])) OR (glucagon like peptide 1 receptor agonists[MeSH Terms])) OR (semaglutide[MeSH Terms])) OR (exenatide[MeSH Terms])) OR (liraglutide[MeSH Terms])) OR (rGLP-1 protein[MeSH Terms])) OR (tirzepatide[MeSH Terms])) OR (lixisenatide[MeSH Terms])) OR (dulaglutide[MeSH Terms])) OR (xultophy[MeSH Terms])) OR (efpeglenatide[MeSH Terms])) OR (Cotadutide[MeSH Terms])) OR (Danuglipron[MeSH Terms])) OR (retatrutide[MeSH Terms])) OR (mazdutide[MeSH Terms])) OR (orforglipron[MeSH Terms])) OR ((((((((((((((((((((((((((((((((((((((((((((((((((((((((((((((((((((((((Incretin mimetic[Title/Abstract]) OR (glucagon like peptide receptor agonist[Title/Abstract])) OR (glucagon like peptide 1 receptor agonist[Title/Abstract])) OR (GLP receptor agonist[Title/Abstract])) OR (GLP1 receptor agonist[Title/Abstract])) OR (GLP 1 receptor agonist[Title/Abstract])) OR (Glucagon like peptide RA[Title/Abstract])) OR (Glucagon like peptide 1 RA[Title/Abstract])) OR (GLP RA[Title/Abstract])) OR (GLP1 RA[Title/Abstract])) OR (GLP 1 RA[Title/Abstract])) OR (GLP1R agonist[Title/Abstract])) OR (glucagonlike peptide[Title/Abstract])) OR (glucagonlike peptide 1[Title/Abstract])) OR (glucagonlike peptide receptor[Title/Abstract])) OR (glucagonlike peptide receptor agonist[Title/Abstract])) OR (glucagonlike peptide RA[Title/Abstract])) OR (glucagonlike peptide 1 receptor[Title/Abstract])) OR (glucagonlike peptide 1 receptor agonist[Title/Abstract])) OR (glucagonlike peptide 1 RA[Title/Abstract])) OR (glucagonlike peptide analogue[Title/Abstract])) OR (glucagonlike peptide 1 analogue[Title/Abstract])) OR (glucagon like peptide analogue[Title/Abstract])) OR (glucagon like peptide 1 analogue[Title/Abstract])) OR (GLP1 analogue[Title/Abstract])) OR (GLP 1 analogue[Title/Abstract])) OR (Semaglutide[Title/Abstract])) OR (Exenatide[Title/Abstract])) OR (Liraglutide[Title/Abstract])) OR (Albiglutide[Title/Abstract])) OR (Tirzepatide[Title/Abstract])) OR (Lixisenatide[Title/Abstract])) OR (Dulaglutide[Title/Abstract])) OR (Ozempic[Title/Abstract])) OR (Rybelsus[Title/Abstract])) OR (Wegovy[Title/Abstract])) OR (Exendin-4[Title/Abstract])) OR (Byetta[Title/Abstract])) OR (Bydureon[Title/Abstract])) OR (Saxenda[Title/Abstract])) OR (Victoza[Title/Abstract])) OR (exenatide extended release[Title/Abstract])) OR (Xultophy[Title/Abstract])) OR (Eperzan[Title/Abstract])) OR (Mounjaro[Title/Abstract])) OR (Zepbound[Title/Abstract])) OR (Lyxumia[Title/Abstract])) OR (Adlyxin[Title/Abstract])) OR (LixiLan[Title/Abstract])) OR (Suliqua[Title/Abstract])) OR (Trulicity[Title/Abstract])) OR (Efpeglenatide[Title/Abstract])) OR (Pegapamodutide[Title/Abstract])) OR (Cotadutide[Title/Abstract])) OR (retatrutide[Title/Abstract])) OR (petrelintide[Title/Abstract])) OR (mazdutide[Title/Abstract])) OR (orforglipron[Title/Abstract])) OR (Danuglipron[Title/Abstract])) OR (Efpeglenatid[Title/Abstract])) OR (Pegapamodutid[Title/Abstract])) OR (Cotadutid[Title/Abstract])) OR (retatrutid[Title/Abstract])) OR (petrelintid[Title/Abstract])) OR (mazdutid[Title/Abstract])) OR (Semaglutid[Title/Abstract])) OR (Exenatid[Title/Abstract])) OR (Liraglutid[Title/Abstract])) OR (Albiglutid[Title/Abstract])) OR (Tirzepatid[Title/Abstract])) OR (Lixisenatid[Title/Abstract])) OR (dulaglutid[Title/Abstract]))) AND (“hidradenitis suppurativa”[MeSH Terms] OR “hidradenitis suppurativa”[MeSH Terms] OR “hidradenitis suppurativa”[MeSH Terms] OR (“hidradenitis suppurativa”[Title/Abstract] OR “suppurative hidradenitis”[Title/Abstract] OR “acne inversa”[Title/Abstract] OR “verneuil disease”[Title/Abstract] OR “verneuils disease”[Title/Abstract] OR “verneuil s disease”[Title/Abstract] OR “inverse acne”[Title/Abstract] OR “hidradenitis suppurative”[Title/Abstract] OR (((“pyoderma”[MeSH Terms] OR “pyoderma”[All Fields] OR “pyodermia”[All Fields]) AND “fistulans”[All Fields]) AND “significa”[Title/Abstract]) OR “apocrine acne”[Title/Abstract]))
